# The Prevalence of Hjortsjo Crook Sign of Right Posterior Sectional Bile Duct and Bile Duct Anatomy in ERCP

**DOI:** 10.1155/2017/2532610

**Published:** 2017-07-12

**Authors:** Hanan M. Alghamdi, Afnan F. Almuhanna, Bander F. Aldhafery, Raed M. AlSulaiman, Ahmed Almarhabi, Abdulaziz AlQurain

**Affiliations:** ^1^Department of Surgery, King Fahd Hospital of the University, University of Imam Abdulrahman Bin Faisal College of Medicine, Dammam, Saudi Arabia; ^2^Department of Radiology, King Fahd Hospital of the University, University of Imam Abdulrahman Bin Faisal College of Medicine, Dammam, Saudi Arabia; ^3^Department of Internal Medicine, King Fahd Hospital of the University, University of Imam Abdulrahman Bin Faisal College of Medicine, Dammam, Saudi Arabia

## Abstract

**Aim:**

The frequency of the Right Posterior Sectional Bile Duct (RPSBD) hump sign in cholangiogram when it crosses over the right portal vein known as Hjortsjo Crook Sign and the bile duct anatomy are studied. Knowledge of the implication of positive sign can facilitate safe resection for both bile duct and portal vein.

**Methods:**

Prospectively, we included 237 patients with indicated ERCP during a period from March 2010 to January 2015.

**Results:**

The mean age (±SD) and male to female ratio were 38.8 (±19.20) and 1 : 1.28, respectively. All patients are Arab from Middle Eastern origin, had biliary stone disease, and underwent diagnostic and therapeutic ERCP. Positive Hjortsjo Crook Sign was found in 17.7% (42) of patients. The sign was found to be equally more frequent in Nakamura's RPSBD anatomical variant types I, II, and IV in 8.4% (20), 6.8% (16), and 2.1% (5), respectively, while rare anatomical variant type III showed no positive sign.

**Conclusion:**

Hjortsjo Crook Sign frequently presents in RPSBD variation types I, II, and IV in our patients.

## 1. Introduction

The anatomy of the bile duct (BD) is resembling that of the portal system and liver segments. Based on the literature, the proportion of biliary anatomical variations varies between 28% and 43%. Most of hilar bile ducts anatomical variations stem from different Right Posterior Sectional Bile Duct (RPSBD) origin [1, 2].

Shimizu's operative series showed that the RPSBD is most commonly supraportal in 84%, infraportal in 13%, and rarely a combination of both in 3% (the segment VII duct being supraportal and segment VI being infraportal) [3]. Furthermore, Nakamura's operative series report the supraportal RPSBD to be most common in BD variant type I (65%, the classic form where the RPSBD and the anterior sectional BD join to form a single right hepatic duct), type II (9.2%, the RPSBD joins the confluence, forming trifurcation), and type IV (15.8%, the RPSBD joins the left hepatic duct), whereas the infraportal RPSBD is reported to be most common in type III (8.3%) and that of the combination in type V (1.7%) [4].

The recognition of the hump appearance in animal cholangiogram being due to supraportal upward course of the RPSBD was first reported by Hjortsjo Crooks in 1951 [5]. The sign can be positive for the supraportal type BD in the classic Nakamura type I, II, or IV. Recognition of Hjortsjo Crook Sign (HCS) in ERCP can enrich our preoperative knowledge of biliary anatomical variation; their precise delineation and anticipation for technical modifications are vital to achieving safe curative liver resection [3] and liver transplantation [4, 6–8] and to avoiding biliary injury in common general surgical procedure like cholecystectomy [9–11].

Our study describes the characteristics of HCS of the RPSBD anatomy in relation to the right portal vein (RPV) among Middle Eastern population using ERCP cholangiogram. To date, the relation of the different anatomical variation of the RPSBD to the RPV based on HCS has never been examined before in humans.

## 2. Materials and Methods

### 2.1. Patients and Methods

This prospective study was carried out during the period from March 2010 to January 2015. We prospectively included 237 consecutive patients who have undergone ERCPs fulfilling the inclusion criteria of being from adult age group (above 18 years old), being from Middle Eastern origin, and having the underlying condition of biliary disease only. Furthermore, patients with complete imaging study and without any prior history of liver resection or biliary instrumentation were considered also as inclusion criteria, while criteria like incomplete study, previous liver surgery, and previous liver transplantation were considered as exclusion criteria. Relevant demographic and laboratory data are obtained and depicted in Tables 1 and 2. The ERCP cholangiogram was reviewed by two radiologists separately. Further filling and focused image in ERCP were done if needed during the procedure (with standard ERCP technique using semiprone position); then the biliary anatomy and the HCS are interpreted by two different radiologists.

This research is supported by the University of Imam Abdulrahman Bin Faisal (formerly known as University of Dammam) (Institutional Research Board: 201054); accordingly, the ethics approval was obtained and informed consent was weaved.

### 2.2. Statistical Analysis

Data analyses included descriptive statistics computed for continuous variables, including means, standard deviations (SD), and minimum and maximum values as well as 95% CI. Frequencies were used for categorical variables. In this study, there was no attempt at imputation for missing data. For all tests, significance is defined as *p* < 0.05 (95% confidence interval). All statistical analyses were done using SPSS 12 (Chicago, Illinois, USA).

## 3. Result

Most of our patients are from youthful age groups due to general young population with mean age (±SD) of 38.8 (±19.20). The predominance of female gender (male to female ratio was 1 : 1.28) reflects the prevalence of the biliary disease in females (Table 1). All patients are Arab from Middle Eastern origin, had biliary stone disease, and underwent diagnostic and therapeutic ERCP. Biochemical data for all patients is in line with biliary stone complications (Table 2).

Anatomical variation of RPSBD based on Nakamura's classification is depicted in Table 3 and showed predominance of types I, II, and IV to be 61.1%, 17.8%, and 16%. Type III RPSBD variant was rare in our population (3.4%) while type V is not detected. Only four patients (1.7%) had undetermined RPSBD anatomical variation.

Most importantly, positive HCS was detected more frequently among patients with type I RPSBD anatomy, in 20 patients (8.4%). The second commonest occurrence of positive HCS was found in type II RPSBD variant, in 13 patients (6.8%). On the other hand, a rare type III RPSBD anatomy was found in only 8 patients and all were found to have negative HCS. One more positive HCS was found in undermined type of RPSBD (0.4%). The presence of positive HCS is depicted in Table 3.

## 4. Discussion

Knowledge of details of hepatobiliary anatomy is vital while performing complex surgical procedures such as hepatobiliary surgeries or liver transplant. This is particularly essential when it comes to anatomic areas with high rates of variations. Multiple biliary orifices in hilar transection plane requiring complex reconstruction are as common as 26% in Ohkubo's and 39.6% in Kasahara's operative series, requiring complex hilar dissection [1, 6]. Hence, the extensive preoperative imaging studies to determine the bile ducat anatomical variant are of paramount importance.

In typical biliary duct course, the lateral hepatic bile duct supplying segments VI and VII and the paramedian hepatic bile duct supplying segments V and VIII reunite to form the right hepatic bile duct (RHD). However, it has been reported that this kind of modal disposition is only associated with 57% of the cases [12]. Many anatomic variations of the convergence of biliary ducts are reported, where the RHD may join the main hepatic duct below the normal confluence level (anterior region in 9% of cases and posterior region in 16% of cases). However, there are situations where the right anterior and posterior segmental bile ducts do not form the right hepatic duct and in 6% to 9% of the cases the right anterior segmental duct joins the left hepatic duct while in 7% to 14% of the cases the anterior segmental duct joins the hilar confluence and forms and three-branch type hilar confluence (c); similarly, in 9% to 27% cases, the posterior segmental duct joins the left hepatic duct [12–14].

To determine the specific anatomical variations, several studies have been conducted using different modalities like cadaveric research [15], intraoperative cholangiogram [16, 17], or imaging such as ultrasonography [18] and magnetic resonance cholangiography [19, 20]. On the other hand, ERCP is the standard technique in this field and provides, if done properly, a detailed anatomy of the extrahepatic and the intrahepatic biliary anatomy as well [21].

Due to expansion and advancement in surgical intervention in hepatobiliary conditions and transplant, this area has moved from anatomy books and being an area of clinical research to fulfilling practical needs [22]. Previous studies based on West or Far East patient population have reported anatomic variants of hepatobiliary system detected by intraoperative cholangiography, MRCP (magnetic resonance cholangiography), or ERCP [23–26].

The ERCP procedure was used in this study to document the variant biliary anatomy of the RPSBD and to investigate the usefulness of positive HCS in delineation of the RPSBD in relation to right postal vein as demonstrated in cholangiogram obtained through ERCP.

To our knowledge, this is the first study to examine the relationship between HCS and the various patterns of the RPSBD variable anatomy in humans and the reported data can be better representative database for our population.

The anatomical variations of RPSBD are similar to the international published data with predominance of types I and II (61.1% and 17.8%, resp.). However, we found more frequently type IV (16%) than type III (3.4%) (Table 3). Low incidence of type III in which the RPSBD drains into the common bile duct was recognized as “cysticohepatic ducts” and its prevalence is very low (1-2%). Our findings are consistent with other studies that reported only 2% of the cases where the RPSBD drained into the cystic duct. Prior information on HCS will help in dealing with the anatomical abnormality especially in the context of RPSBD, where the cystic duct can be ligated between the gallbladder and the point at which the duct joins [27, 28].

We found HCS to be positive in 17.7% of the patients and more frequently positive in types I, II, and IV RPSBD anatomy in 8.4%, 6.8%, and 2.1%, respectively. On the other hand, in a rare type III RPSBD anatomy, all were found to have negative HCS. One more positive HCS was found in undermined type of RPSBD (0.4%) (Table 3).

A possible limitation of this study was that it did not evaluate the patterns of HCS in a healthy population [29]. Irrespective of that, our data may be more representative of the general population than data from other populations.

In conclusion, our study reveals that types I, II, and IV RPSBD anatomical variation is more commonly showing positive HCS than any other type. Prior knowledge of this sign is essential to achieve curative resection in some cases with an abnormal pattern of the RPSBD. Since elusive knowledge of the biliary anatomy at hepatic hilum in hepatobiliary surgery may easily lead to postoperative biliary complication [4, 8], preoperative recognition as well as intraoperative understanding of the RPSBD is apparently important for safe and curative resection in patients with aberrant biliary system. Likewise, avoiding biliary complications for both donor and recipient in living donor liver transplantation (LDLT) is critical to achieving safety for both. One of the major biliary complications in patients undergoing LDLT is the anatomical limitations contributed by multiple tiny bile ducts and the differential blood supplies. Recognizing these anomalies with the aid of HCS preoperatively, this may result in dramatic drop in the incidence of biliary complications and improve outcome and selection of donors in LDLT in our populations. Although in LDLT the donor will not undergo ERCP as standard evaluation test, the knowledge of the importance of HCS can be useful for comparison of data obtained from less sensitive modalities like magnetic resonance cholangiopancreatography (MRCP).

## Figures and Tables

**Table 1:** Patient demographic data.

	*N* = 237
*Age:*	
(i) Mean (±SD)	38.8 (19.20)
(ii) Median (range)	34.033 (18–97)
*Gender*	
Male	104
Female	133
M : F ratio	1 : 1.28
*Nationality*	
Saudi	199
Others (Middle Eastern)	37
*Total*	237

*N*: number.

**Table 2:** Biochemical profile of all patients.

Variables	Normal ranges	*N* = 237
		Mean ± SD
T Bili	(0.1–1.0)	8.7655 ± 21.78339
D Bili	0.0–0.4	6.9978 ± 17.24988
Alkaline phosphatase	50–140	254.0222 ± 224.22206
PT	11–14	12.6705 ± 2.45859
GGTP	5–85	269.8923 ± 325.76886
Albumin	3.5–4.8	3.7143 ± 3.64814
WBC	4–11	8.4414 ± 3.75207
Platelet	140–440	285.0127 ± 138.17845
Amylase	25–125	218.7683 ± 484.17567
Lipase	4–24	1348.9000 ± 4559.71331

T Bili: total bilirubin; D Bili: direct bilirubin; PT: prothrombin time; *N*: number.

**Table 3:** Comparative evaluation of different types of Hjortsjo Crook Sign.

RPSBD anatomical variant^§^	Positive HCS *N* (%)	Negative HCS *N* (%)	Total
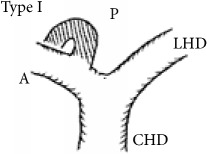	20 (8.4)	125 (52.7)	*145* *(61.1)*
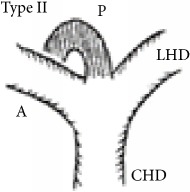	16 (6.8)	26 (11)	*42* * (17.8)*
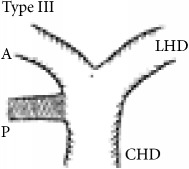	0	8 (3.4)	*8* * (3.4)*
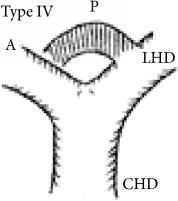	5 (2.1)	33 (13.9)	*38* * (16)*
Type V Mixed type	0	0	*0*
Undetermined	1 (0.4)	3 (1.3)	*4* * (1.7)*
*Total*	*42 (17.7)*	*195 (82.3)*	

RPSBD: Right Posterior Sectional Bile Duct. ^§^Nakamura's classification of RPSBD. LHD: left hepatic duct. CHD: common hepatic duct. A: Right Anterior Sectional Bile Duct. P: Right Posterior Sectional Bile Duct. *N*: number. HCS: Hjortsjo Crook Sign. Data are frequency counts (percentage of total).
